# Adapting a Youth Sexual Violence Prevention Logic Model and Evaluation to Asian American and Pacific Islander Youth in Iowa: a Case Study

**DOI:** 10.1007/s11121-025-01851-6

**Published:** 2025-11-20

**Authors:** M. Yusef, K. Bailly, B. Lu, H. Haines, A. Sursely, M. L. Goedken, R. Beltran, R. A. Afifi

**Affiliations:** 1 Monsoon Asians and Pacific Islanders in Solidarity, Des Moines, IA USA; 2 Department of Community and Behavioral Health, College of Public Health, University of Iowa, 145 N Riverside Dr., Iowa City, IA 52242 USA; 3 Prevention Research Center for Rural Health, University of Iowa, Iowa City, IA USA; 4 Iowa Department of Health and Human Services, Des Moines, IA USA

**Keywords:** Asian American and Pacific Islander (AAPI), Youth violence prevention, Culturally specific logic models, Academic-community partnership, Rape Prevention and Education (RPE)

## Abstract

Asian American and Pacific Islanders (AAPI) youth report a high prevalence rate of youth violence. Logic models that guide violence prevention programming are often not culturally specific and may not address the unique structural/social drivers of youth violence in minoritized communities. Monsoon has received Rape Prevention and Education (RPE) funding from the Iowa Department of Health and Human Services (Iowa HHS) since 2007 through a competitive application process. The specific aim of Monsoon’s Youth Sexual Violence Prevention Program (YVPP) is to “prevent gender-based violence by educating and empowering peers through outreach and workshops.” In response to growing awareness at Iowa HHS about the limitations of the State RPE programmatic logic model in addressing AAPI-specific context for youth violence, Monsoon received additional RPE funds to establish the first culturally specific RPE logic model in Iowa. This initiative aims to fill a gap in culturally relevant evaluation resources. This paper describes the academic-community partnership initiated in 2023 that guided the participatory development of the culturally specific logic model and evaluation plan, for youth violence prevention in AAPI communities. Monsoon partnered with staff and faculty from the Center for Disease Control and Prevention (CDC)-funded Prevention Research Center for Rural Health (PRC-RH) in this work. PRC-RH faculty and staff reviewed documents and interviewed Monsoon leaders and youth staff. An AAPI-specific youth staff logic model and evaluation research questions were developed using a community-based research approach. Based on these, a mixed-methods evaluation plan was outlined. This case study highlights the critical importance of community-engaged research and practice to develop culturally specific logic models and evaluation designs for youth sexual violence prevention in the AAPI community.

**Supplementary Information:**

The online version contains supplementary material available at 10.1007/s11121-025-01851-6.

## Background

Violence is a leading cause of morbidity and mortality for young people aged 10–24 years in the USA. Drivers of youth violence include social and structural factors (Armstead et al., 2021; Curtin & Garnett, 2023; David-Ferdon et al., 2021; National Sexual Violence Resource Center, 2018), such as “ideologies, systems, institutions, and policies that utilize power to create and perpetuate social, political, and economic environments that harm some groups of people while empowering or privileging others” (Wendel et al., 2021). As a result, youth violence occurs disproportionately among youth of color (Nation et al., 2021). Within the broader nomenclature of violence, sexual violence is a distinct form of harm: 25% of girls and 17% of boys are sexually abused before 18 years of age, and nearly 50% of all students in grades 7–12 have experienced sexual harassment (National Sexual Violence Resource Center, 2018).

This paper focuses on Asian American and Pacific Islanders (AAPI). AAPI is an umbrella political term used in policy and advocacy to unite people who identify with that race: 24 million people across all 50 states plus US Territories, representing 75 countries (AAPI Equity Alliance, 2025). Structural factors play a significant role in perpetuating violence within AAPI communities, especially sexual violence. The USA has a long history of racism and violence against AAPI, through colonialism, normative ideas around oppression and exploitation, and nineteenth- and twentieth-century immigration and naturalization policies (Kulkarni, 2023; Morey et al., 2020). The exotification, fetishization, and hypersexualization of women, as well as the model minority myth and the emasculation of males, all specifically contribute to sexual violence (Lim et al., 2023).

The relationship between structural drivers and sexual violence within AAPI communities is complex, in part due to the lack of disaggregated data. This absence of detailed information contributes to the de-contextualization of the diverse subgroups encompassed by the term AAPI (Raman et al., 2024). These structural drivers also encompass US military sexual violence, which includes “the impact of the military on civilian victims, individuals engaged in the sex trade, native populations residing near military installations, and the enduring legacy of trauma” (NAPIESV, 2023). As previously noted, AAPI communities are not monolithic, and experiences of sexual violence can vary significantly across different subgroups.

Studies reporting rates of sexual violence among AAPI adults are limited. A scoping review of non-partner “Sexual Violence among Asian American, Native Hawaiian, and Pacific Islander Adults” (AANHPI) found two studies that indicated rates of sexual violence were lower among API women than all other races, but also noted the lack of data on prevalence for AANHPI communities (Lim et al., 2023). One research study indicated that Native Hawaiian and Pacific Islander women experience two to three times more sexual assault than their White and Asian American counterparts (Crisanti et al., 2011). According to the Iowa HHS, the rate of emergency department visits for sexual violence among the Asian or Pacific Islander communities was 19.7 per 100,000 in 2023. Furthermore, it is trending upward with the rate increasing from 14.9 per 100,000 in 2021. From this incident data, we know sexual violence is prevalent in the AAPI community.

Prevalence rates of sexual violence for AAPI youth are equally lacking, despite reports that indicate increasing, and perhaps disproportionate rates of violence (Lai, 2008). Recent studies conducted in school settings have shown API youth experience high levels of hate speech, bullying, teen dating violence, and inequitable treatment (Gee et al., 2024; Lu et al., 2024). In Iowa, although AAPI youth make up less than 1% of the student population under 18 years of age (State Data Center of Iowa, 2024), they account for over 4.3% of all student victims of violence (Iowa Department of Education, 2023). This is an underrepresentation as it does not include AAPI students who identify as multi-racial or those who do not report the violence they experience at school. Additionally, Youth Behavioral Risk Factor survey data from Hawaii found that up to 15% of API youth reported being “forced to do sexual things by someone they were dating or going out with in the past 12 months” (Asian Pacific Institute on Gender Based Violence, 2020). Furthermore, an online survey across 33 American universities found that approximately 10% of Native Hawaiians and other Pacific Islanders college students reported “experiencing penetration or sexual touching without voluntary agreement” (Asian Pacific Institute on Gender Based Violence, 2020).

The Center for Disease Prevention and Control’s (CDC’s) Rape Prevention Education (RPE) program aims to prevent the first-time occurrence of sexual violence—both victimization and perpetration—by providing funding to state and territorial health departments. These departments collaborate with state Sexual Assault Coalitions to plan, implement, and evaluate public health strategies and activities focused on sexual violence prevention (Centers for Disease Control and Prevention, 2025). RPE prevention programming is subcontracted to community-based organizations in Iowa and other states (Centers for Disease Control and Prevention, 2025). The CDC promotes a public health approach and the social-ecological model to guide RPE programming. This framework emphasizes the need to address the drivers of violence across multiple levels, and encourages health departments to use the Sexual Violence Prevention: Resources for Action toolkit (Basile et al., 2016), which compiles the best available evidence for effective prevention strategies. Several systematic reviews of sexual violence prevention programs have been published since 2016 (Ellsberg et al., 2018; Finnie et al., 2022; Miele et al., 2023; Mujal et al., 2021; National Sexual Assault Resource Center – NSARC, 2018; Piolanti et al., 2022; Tibbels et al., 2024). Among these, including the CDC’s guidance (Basile et al., 2016), only the NSARC (2018) provides guidance for culturally relevant effective services to sexual assault survivors.

To support program development and evaluation, the CDC’s RPE program developed a general logic model for grantees. Logic models are essential tools that articulate a program’s theory of change and “illustrates the association between your program’s resources, activities, and intended outcomes” (Centers for Disease Control & Prevention, 2024). The CDC’s RPE logic model reflects an ecological framework, highlighting the importance of environmental-, community- (e.g., social norms), organizational- (e.g., school), and individual-level changes. While this model is useful in guiding grantee objectives and activities, it represents the overarching theory of change for the national RPE program and is not tailored to specific community contexts. As Meyer et al. (2022) emphasize, programmatic theories of change and logic models must reflect the unique circumstances of the communities they serve. To be most effective, these tools should be developed *with* communities, not *for* them, ensuring that structural drivers are meaningfully addressed, thus avoiding further marginalization. Echoing this point, Nation et al. (2021) assert that youth violence prevention efforts must explicitly engage with structural drivers to succeed.

The Iowa HHS receives CDC RPE funding to strengthen the state’s public health infrastructure for sexual violence prevention by planning, implementing, and evaluating evidence-based and community-informed programs. RPE funds are used to develop a State Action Plan in collaboration with the Iowa Coalition Against Sexual Assault, the University of Iowa Injury Prevention Research Center, and the Iowa Violence Prevention Data Steering Committee. Additionally, RPE funds training, technical assistance, evaluation, and data to action to prevent the first-time occurrence of sexual violence among key impacted communities. A portion of funds is awarded to community-based organizations to implement violence prevention programs that address social and environmental factors identified in the statewide programmatic logic model, State Action Plan, and Evaluation Plan. One of the competitively funded recipients of these prevention activities is Monsoon Asians and Pacific Islanders in Solidarity (Monsoon). Monsoon’s mission is to end all forms of gender-based violence and build healthy communities through transformative justice and social change. Monsoon provides direct services to AAPI victims/survivors of domestic violence, sexual assault, and human trafficking as well as education and outreach to the wider community (Monsoon Asians and Pacific Islanders in Solidarity). Monsoon’s RPE program focuses on youth. In 2008, the organization launched the Youth Violence Prevention Program (YVPP) following a community assessment on AAPI communities in Iowa. The assessment highlighted the need for a program that is youth-led, youth-implemented, and youth-evaluated, emphasizing the importance of centering youth voices in violence prevention efforts. The specific aim of Monsoon’s YVPP is to prevent gender-based violence by educating and empowering peers through outreach and workshops. Monsoon’s YVPP is led by youth staff from Des Moines, Iowa. In 2022, rising awareness by Iowa’s RPE program coordinator of the limitations in the state’s existing logic model—particularly its inability to address the specific context of youth violence in AAPI communities—led to Monsoon receiving additional RPE funds. This support enabled the development of Iowa’s first culturally specific RPE logic model. Monsoon partnered with subject matter experts from the Prevention Research Center for Rural Health (PRC-RH) at the University of Iowa College of Public Health to co-develop an AAPI-specific youth violence prevention logic model with a corresponding evaluation plan. This paper describes the academic-community partnership initiated in 2023 that guided the participatory development of Monsoon’s culturally specific logic model and evaluation plan.

## Methods

### Overall Approach

#### Community-Engaged Research and Practice (CEnRP)

The overall approach to the development of the logic model and evaluation plan centered on the principles of community-engaged research and practice. Community engagement is defined as “the process of working collaboratively with and through groups of people affiliated by geographic proximity, special interest, or similar situations to address issues affecting the well-being of those people” (ATSDR’s community engagement playbook 2021, 2021). The continuum of CEnRP has “no community engagement” on one side and “community-driven/led” on the other (Key et al., 2019). Since this adaptation request was initiated by Monsoon, we consider this work to be community driven/led. As a result, the PRC-RH’s academic evaluation team’s role is to support community-identified needs (Key et al., 2019). Specific to youth, Youth Participatory Action Research (YPAR) is a community-based participatory research approach that considers youth to be experts who guide the identification of needs and strengths and use those to develop and implement interventions (Ozer et al., 2020). A YPAR approach was implemented in the adaptation of the logic model and development of the evaluation plan. Engaging AAPI youth in developing logic models centers their lived experience and begins to reverse dominant narratives and power dynamics (Lee et al., 2022; Sprague et al., 2019).

### Preparatory Work

#### Monsoon Values

Interventions are often seen as incorporating “sets of values” (Hawe, 2015). Monsoon’s core values guide the design of all their interventions, including the YVPP. These include Anti-Imperialism/Anti-Colonialism, Decolonization, Social Justice/Social Change, Anti-Oppression/Intersectionality, Feminist-Activist Framework, Survivor-Centered, Worker-Centered, and Community-Centered. We focused on these values as we developed the logic model and evaluation plan.

#### Review of Documents

Collecting information is often the first step in developing the logic model (McLaughlin & Jordan, 1999). The academic evaluation team (authors RA, HH) began with a review of the following documents: (1) Monsoon’s mission, vision, values, and programming through exploring Monsoon’s website; (2) Monsoon’s YVPP through examining the YVPP submitted grant proposal (*n* = 1), and all progress and annual reports provided to the State RPE program between 2022 and 2023 (*n* = 11); and (3) the State RPE goals and objectives through reading the documentation provided by the State RPE coordinator (*n* = 2).

### Development of the YVPP Overall Logic Model

Logic models often include programmatic inputs, activities, outputs, and short-, intermediate-, and long-term outcomes. Our visualization of the logic model for the Monsoon program focuses specifically on the outcomes, and links them to activities at a later stage. As noted above, the Monsoon YVPP is led and implemented by Monsoon hired youth leaders and staff (YLS). These Monsoon YLS conduct activities in the community with other AAPI youth (Supplemental Appendix A for examples of the activities conducted by YLS). The logic model for the YVPP was developed through the following steps:

1. *Understanding the drivers of AAPI youth sexual violence*: The next step of logic model development (after the preparatory work) is often about carefully defining the problem and understanding its context (McLaughlin & Jordan, 1999). The academic evaluation team interviewed the executive director (ED) of Monsoon (author MY) in a meeting that lasted 1.5 h to understand the YVPP’s culturally identified drivers of youth violence in the AAPI community. Starting with youth violence, we queried immediate, intermediate, and distal/structural risk and protective factors considered by the YVPP. This led to the development of a draft logic model of drivers.
2. *Focus group discussion* (FGD) with YVPP leaders (authors KB and BL) and staff: The academic evaluation team subsequently conducted one FGD with YVPP leaders and staff (YLS) to (i) understand their vision and dreams for the YVPP, (ii) receive feedback on the draft logic model of drivers developed after the discussion with the executive director of Monsoon and finalize it, and (iii) begin to link the activities conducted within the YVPP to the risk and protective factors within the logic model (Supplemental Appendix B includes the agenda for this meeting, and the specific questions asked). The FGD was 1 h in length. All YLS were invited, and all but two were able to attend. The discussion in this meeting highlighted an unexpected outcome: the impact of implementing the YVPP on the YLS themselves.
3. *Analysis of the interview and FGD:* We used single-case study narrative analysis (Rabu & Binder, 2025; Greenhalgh, 2016; Thompson & Kreuter, 2014) to make meaning out of the insight and stories gathered. In addition, the CEnRP approach allowed for meaningful member checking at every stage of the process of development of the logic model and evaluation plan (McKim, 2023).
4. *“AHA”* *moment and feedback to Monsoon ED and Iowa RPE Coordinator:* FGD results led to an AHA moment for the academic evaluation team: the realization that there were two interventions imbedded in the YVPP—one intervention focused on YLS and the second on AAPI community youth. We reached out to the Monsoon ED and Iowa RPE Coordinator to share our realization and discuss next steps. A decision was made to develop a second logic model for the YLS and focus on this second logic model for the evaluation plan. We decided to develop two logic models instead of imbedding them into each other, as the focus of the YLS logic model was strength-based, whereas that for community youth was more deficit- based.

### Development of the YLS Logic Model

1. *Drafting the YLS* *logic model*: Results of the FGD were used to develop a second logic model focused on the risk and protective factors.
2. *Feedback from the Monsoon ED and YLS on the second logic model:* We shared the second logic model with the ED and YLS and made minor edits based on their feedback.
3. *Linking intervention activities for YLS to the logic model drivers:* We created a template for youth leaders to complete linking intervention activities they received to the YLS logic model.

### Development of the YLS Evaluation Plan

To develop the YLS evaluation plan, the academic team began by discussing the focus of the evaluation (Kidder et al., 2024) with Monsoon ED. Monsoon’s RPE YLS evaluation purpose—linked to the overall Iowa State RPE program evaluation purpose—is to improve the overall functioning/effectiveness of Monsoon’s RPE YLS program. Evaluating the program implementation will result in a more comprehensive understanding of what program elements are working well/as planned and which areas need improvement/additional efforts. Evaluation efforts will consist of both process- and outcome-oriented evaluation questions that enable understanding of what outcomes the programming is achieving and what processes and practices contributed to those outcomes. In line with this purpose, a decision was made to focus the evaluation both on the process of implementation of activities and the impact of activities on YLS.

Once this decision on focus/scope of evaluation was made, evaluation research questions were co-developed in a meeting with Monsoon ED, YLS, and the academic evaluation team. The research questions include evaluating the program’s implementation and program outcomes. We then began to develop the evaluation plan guided by the CDC’s evaluation framework (Kidder et al., 2024). This framework includes six steps: (a) assess context, (b) describe the program, (c) focus the evaluation questions and design, (d) gather credible evidence, (e) generate and support conclusions, (f) act on findings. Steps (a) and (b) are described as part of the logic model development. In what follows, we focus on step (c). Steps (d)–(f) will be part of the report once the evaluation plan is put into practice. In line with the participatory approaches (CEnRP and YPAR) we implemented throughout this process, the CDC evaluation framework emphasizes the importance of “interest holders” engagement as an iterative process throughout the evaluation.

However, this participatory process also emphasized the importance of centering Monsoon’s values. The academic evaluation team thus explored emancipatory frameworks to support the CDC’s evaluation framework. In discussions with Monsoon’s ED, three were selected to ensure we did not lose sight of those values:

(i) Intersectional approach to evaluation (Kearney et al., 2018): This approach underscores the intersectional forms of discrimination (AAPI, gender, nationality, disability, age, etc.) and confronts power dynamics.
(ii) Feminist evaluation (Crupi & Godden, 2023; Sielbeck-Bowen et al., 2002): Components include community driven, led, and owned; integrating collective action; focusing on intersectionality and power; building consciousness, skills, and capabilities; and ensuring safety and collective care.
(iii) Anti-Colonial Culturally Responsive Evaluation Framework (ACREF) (Jordan & Hall, 2023): The ACREF combines decolonizing frameworks with culturally responsive evaluation. The ACREF approach challenges Westernized methodology and centers Indigenous knowledge production. ACREF is “an intentionally political, resistance-based stance, (and) prioritize(s) issues of culture and justice” (Jordan & Hall, 2023). ACREF also emphasized a strength-based (rather than deficit-based) narrative of community.

The academic evaluation team then developed a community-engaged mixed-methods evaluation plan that was in line with previous research in violence prevention (Banyard et al., 2023). Culturally specific process- and outcome-oriented questions were included, along with suggested tools, audiences, and timing of evaluation.

## Results

We describe below the various results/outputs of the process of developing the logic model(s) and evaluation plan.

### Understanding the Drivers of AAPI Youth Violence

**Fig. 1:**
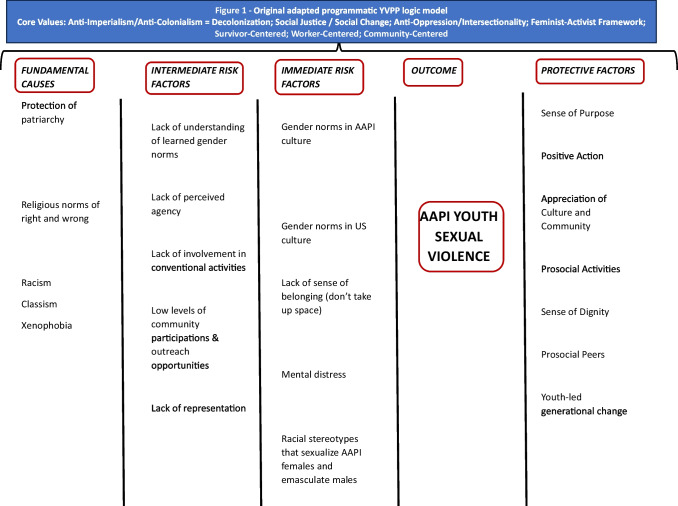
Original Adapted Programmatic Logic Model

This logic model was constructed through conversations with the Monsoon ED and the YLS. Its focus was on the context and drivers that lead specifically to experiencing youth violence in the AAPI community. The academic evaluation team did not prompt for structural or social drivers, nor for drivers at the ecological levels (Centers for Disease Control and Prevention, 2024). However, as is evident in Fig. 1, these structural and ecological levels were clearly vocalized by the Monsoon ED and YLS. In keeping with the ACREF framework that uplifts different ways of knowing, we took the community-identified drivers as evidence, without searching for peer-reviewed publications confirming these, although for most, if not all, such positivistic evidence can be found. The identified context/drivers were categorized into immediate and intermediate risk factors and fundamental causes (Link & Phelan, 1995). Immediate risk factors included gender norms in the AAPI culture and the US culture. Intermediate risk factors included lack of representation, lack of community participation, and a lack of perceived agency. The fundamental causes included racism, xenophobia, and the protection of patriarchy. Figure 1 also includes protective factors that prevent the experience of youth violence in the AAPI community, including youth-led intergenerational change. As noted in the “Methods” section, we did not link Monsoon’s YVPP community activities (Supplemental Appendix A) to the risk factors/fundamental causes of this logic model, nor did we pursue the development of an evaluation plan for this logic model.

### Youth Leaders and Staff (YLS) Logic Model

**Fig. 2:**
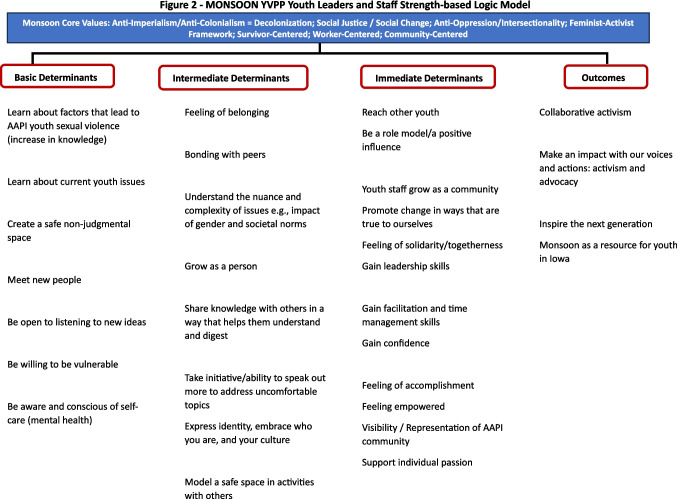
Monsoon YVPP Youth Leaders and Staff Strength-based Logic Model

The FGD with YLS made clear that their engagement as YLS and their preparatory work to be able to implement the YVPP activities with the AAPI community was an intervention for them. The YLS highlighted their excitement and growth in critical consciousness during this process, the skills that they gained, and their feelings of agency, empowerment, and representation. Post FGD dialogue between the academic evaluation team and Monsoon’s ED and the RPE coordinator, noted the critical importance of this YLS “intervention” in the continuum of the YVPP and in the effectiveness of the YVPP activities with the larger community. As a result, a decision was made to develop a second logic model for the YLS “intervention” and to focus as a start on the evaluation of that component of the larger YVPP. Review of the transcript of the FGD led to the draft YLS logic model which was then shared back with Monsoon’s ED and YSL for review and finalization (Fig. 2).

The logic model for YSL includes basic, intermediate, and immediate drivers that lead to outcomes. At the basic level, YLS learn about factors that lead to AAPI youth sexual violence and about current youth issues. This learning occurs in a space that is nonjudgmental and safe and can happen only if YLS are willing to be vulnerable and open to listening to new ideas. This leads to the intermediate drivers including their own understanding of the nuance and complexity of issues (e.g., impact of gender and societal norms), increasing comfort with their expression of identity and culture, and their own growth. Immediate drivers then include feelings of solidarity with others, being positive role models, and growth as a YLS community. The outcomes of this preparation of YLS included collective activism, inspiring the next generation, and making an impact with their voices and actions. YLS noted the critical importance of an organization like Monsoon, rooted in its values, to be a resource for AAPI youth in Iowa and create the space needed for growth.

### Linking YLS Intervention Activities to the YLS Logic Model

Once Fig. 2 was finalized, youth leaders considered all the activities they participated in to prepare for their role as YLS and linked each to a component of the logic model, and provided an explanation of how the activity resulted in changes in the drivers (Table 1 and Supplemental Appendix C). This task was iterative with the logic model; if any activity did not link to a logic model determinant, then the logic model was adjusted or the activity was removed as a component of the training of YLS. However, no activities were removed as they were each associated with at least one of the drivers in the logic model.

**Table 1:** Exemplar* Training activities implemented with Youth Leaders and Staff (YLS) linked to YLS logic model Instructions given to YLS: For each training activity, list the level of determinant (basic, intermediate, immediate) from the YLS logic model that it is directly tied to impact, and explain how Part I – Learning of YLS —activities involved in their growth as youth leaders and staff

RPE youth leadership training activity	YLS logic model (determinants w/direct impact)	How does it do so?
Training and orientation	Basic: Learn about factors that lead to AAPI youth sexual violence (increase in knowledge) Intermediate: Understand the nuance and complexity of issues, e.g., impact of gender and societal norms	Orientation for youth staff covers an overview of Monsoon’s background, core values, staff roles, programs, the AAPI communities they serve, and a glimpse into the history of AAPI migration in the USA. Additionally, they receive training on sexual assault, domestic violence, and their prevalence within AAPI communities Input from youth staff shapes their training to align with their specific roles and interests during the program. These trainings are also applicable and valuable outside of their work within Monsoon

* The full table is in the supplementary appendix C

### Developing Research Questions

All evaluation design phases were conducted in partnership with Monsoon leadership and YLS. With this in mind, and guided by the YLS logic model, the academic evaluation team developed six main evaluation questions and additional evaluation questions of interest (Supplemental Appendix D). Each has a quantitative and qualitative component. Monsoon leadership and the YLS reviewed, edited, and approved all the research questions.

By linking the evaluation questions to the logic models that were developed through this community-engaged process, principles of shifting power dynamics and centering community (here AAPI) ways of knowing, doing, and being were emphasized—in line with emancipatory approaches to evaluation. In addition, the YLS identified drivers are in fact the critical concepts of these emancipatory evaluation models; such as issues of justice, power, consciousness, capabilities, and collective care.

### Evaluation Design

The overall design of this evaluation will be a case study design. Case study evaluation designs “provide evidence on context, complexity and mechanisms for understanding how, where and why interventions have their observed effects” (Paparini et al., 2020). Contexts are critical factors in the environment that have significant effects on the implementation and outcomes of any intervention. For Monsoon, the specificity of the AAPI cultural characteristics and the AAPI experience in the USA cannot be separated from the development of a violence prevention logic model and program. The approach will include an assessment of the process of implementation and the impact of the activities (Kidder et al., 2024). To meet these dual objectives, a mixed-methods evaluation (Banyard et al., 2023)—quantitative and qualitative questions and methods—was designed.

#### Quantitative Methods

A survey will be fielded with current, future, and past youth staff members, as well as Monsoon leadership. The survey (Supplemental Appendix E) will answer Q. 1–5A (Supplemental Appendix D).

#### Qualitative Methods

Focus group discussions (FGDs) will be conducted with current, future, and past youth staff. Interviews will be conducted with Monsoon leadership. These FGDs and interview guides (Supplemental Appendix F) will answer Q. 1–5B, as well as 5 C, 6A–C, and the extra questions i–iv (Supplemental Appendix D).

For each evaluation question, the following were specified: preliminary measures/indicators, data source/data collection instrument, audiences, timing of evaluation, audience, and data analysis (Supplemental Appendix G). The implementation of the evaluation is planned for 2026. However, Monsoon has experienced loss of grant funding due to the current curtailing of funding for programs focused on health equity and on communities experiencing marginalization. Monsoon continues to apply to other funding opportunities. This evaluation plan can be implemented upon the resumption of prevention infrastructure and funding channels.

## Discussion

Logic models are critical tools to guide program development, implementation, and evaluation (Centers for Disease Control and Prevention, 2024), but to be most effective, they need to consider and adapt to the context in which the programs/intervention will be delivered (Meyer et al., 2022). This paper describes the process of adaptation of the CDC’s RPE logic model to the context of AAPI youth and the subsequent development of an evaluation plan to measure the impact of the intended intervention. The importance of specifying the context in the development of logic models has also been highlighted by others (Castro et al., 2004; Cullen et al., 2016; Evans et al., 2021; McLaughlin & Jordan, 1999; Monroe et al., 2025). Yet, specific examples of contextualized logic models for youth *sexual* violence prevention programs are rare; a recent publication documents a similar co-creation process of logic model and evaluation metrics for a violence prevention program (Roman et al., 2025).

We used a community-driven participatory research and practice approach throughout the process of developing the adapted logic model. The literature on youth participation urges strategies that go beyond tokenism and result in “meaningful youth engagement” (Nwaozuru et al., 2025; Ozer et al., 2020; PMNCH, 2020). A review of methods of participation found that those that encourage input and promote agency and empowerment lead to more meaningful engagement of participants (Willmott et al., 2023). As described in our “Methods” section, YLS were actively involved in the process of developing the logic model and the evaluation design.

Monsoon YLS identified immediate, intermediate, and basic drivers of their ability to gain knowledge and skills to enable their implementation of Monsoon’s YVPP activities with community members. Similarly, structural factors and critical consciousness/awareness of these factors driving youth sexual violence emerged as a main listed determinant, in other analyses of gender-based violence in AAPI communities (Dabby & Yoshihama, 2022). Critical consciousness has also been highlighted as necessary for emancipation in work with AAPI youth more generally (Lee et al., 2022). Social justice youth development hypothesizes that critical consciousness emerges in three stages:

(i) Self-awareness—where they begin to explore identity issues. This emerged in the YLS logic model: express identity, embrace who you are, and your culture.
(ii) Social awareness—the capacity to understand and analyze issues in their own community, identified in the YLS logic model: learn about the factors that lead to APPI youth sexual violence.
(iii) Global awareness—the intersections of struggles of oppression, leading to a sense of purpose and optimism for the future (Ginwright & Cammarota, 2002).

Further, social justice youth programming for Asian American youth led to a sense of empowerment and greater social justice action (Suyemoto et al., 2015) and was similarly experienced by Monsoon’s YLS, for example, noted in the YLS logic model: Make an impact with our voices and actions: activism and advocacy. YLS noted that collective activism is an outcome of their growth as a team.

This project also highlights the importance of partnerships between academic institutions and community-based organizations, made possible by the willingness of the funder to support the ask for additional funding by the grantee to develop an adapted model. As each partner brings their knowledge and skills to bear on an identified issue, the resulting output strengthens all partners. Eleven hindering and 12 facilitating factors to Community-Academic Partnerships were identified through a systematic review (Drahota et al., 2016). Of the facilitators noted, the partnership between Monsoon and the PRC-RH’s academic evaluation team met almost all: we had a good working relationship and had built trust and respect through previous work together. For the work described herein, we had agreed to a shared goal to accomplish, felt the work was mutually beneficial, and were convinced it would lead to a positive impact. We communicated frequently, had well-structured meetings, and experienced no conflict. Of the hindrances noted in the Drahota et al. (2016) review, only the high burden of activities/tasks was present. To conform with the community-engaged approach, we asked the YLS and Monsoon leadership to actively engage with each part of the logic model and evaluation design development, which took time away from their other priorities. Based on our experience, we encourage other academic and community-based organization colleagues to partner together!

Ultimately, the development of the adapted logic model had two main purposes: (1) to document factors at the various levels of the socioecological model influencing APPI youth sexual violence and map YVPP activities onto these factors and (2) to build the capacity and skill of the youth leaders of the YVPP related to monitoring and evaluation. The overall goal of the work is to prevent youth sexual violence perpetration and victimization. Despite the success of the partnership that led to the development of the logic model and evaluation of Monsoon’s YVPP, the current political environment impacts this work significantly (Eriksen et al., 2024). Several of Monsoon’s funding streams have been revoked, and the grants they had planned to pursue for continued work are no longer available. Culturally specific programming is considered to fall under diversity, equity, and inclusion, which is being dismantled in the current administration (White House, 2025). Additionally, terms/words are being monitored for future defunding of certain organizations, words that are often important to organizations that work with communities experiencing disparities. Further, the general increase in anti-immigrant rhetoric (Das, n.d.) amplifies structural factors that lead to sexual violence.

### Limitations

We report on a participatory case study process to adapt a youth sexual violence prevention logic model to AAPI youth and to design a mixed-methods evaluation to measure implementation and impact of the related YVPP. We used a CEnRP approach and chose emancipatory evaluation frameworks that fit the values of the community organization we worked with. Adaptation to other communities experiencing disparities in sexual violence victimization and perpetration will require utilization of frameworks and approaches that most interact with their strengths, needs, and values.

### Practical Implications

This case study provides a model for a community-engaged research and practice approach to the development of contextualized logic models and evaluation—for youth sexual violence prevention (or other health-related outcomes) in communities experiencing disparities resulting from structural drivers. Our experience suggests that co-created tailored logic models are feasible, and lead to clearer programming and evaluation. Further, when developed with youth leaders, they also serve to develop planning and evaluation competencies in the next generation of violence prevention leaders.

### Conclusion

Adapting a youth sexual violence prevention logic model to AAPI youth improves current sexual violence prevention efforts through community-engaged solutions that challenge dominant narratives and foster social justice. It further enhances youth skills in monitoring and evaluation, which can transfer to other programs and activities they implement, ensuring more robust, effective, and potentially sustainable interventions for enhanced well-being and thriving. Culturally and contextually centered and led planning and evaluation begin to flip power dynamics and uplift the promise of social justice and health equity.

## Supplementary Information

Below is the link to the electronic supplementary material.

ESM 1 (PDF 742 KB)
