# Supplementary material for: Adapting a Youth Sexual Violence Prevention Logic Model and Evaluation to Asian American and Pacific Islander Youth in Iowa: a Case Study
Source: Prev Sci. 2025 Nov 20;27(5):762–73. doi: 10.1007/s11121-025-01851-6 (PMC13421215; doi:10.1007/s11121-025-01851-6)
Supplement: Supplementary file 1 — (PDF 742 KB) [file 11121_2025_1851_MOESM1_ESM.pdf]

Supplemental Appendix A

Examples of activities conducted by the Youth Violence Prevention Program Youth Leaders and Staff

**Activity**	**Strategy**
Girls Group at Elementary School	• Promote Social Norms that Protect Against Violence<br>• Teach Skills to Prevent Sexual Violence<br>• Provide Opportunities to Empower and Support Girls and Women<br>• Create Protective Environments
Girls Group and Boys Group at Middle School	• Provide Opportunities to Empower and Support Girls and Women<br>• Create Protective Environments
Boys Group at Elementary School	• Create Protective Environments<br>• Promote Social Norms That Protect Against Violence<br>• Teach Skills to Prevent Sexual Violence
Asian Student Club at High School	• Create Protective Environments<br>• Provide Opportunities to Empower and Support Girls and Women
Monthly Meetings with (City) Public School Coordinators and Community Partners	• Create Protective Environments<br>• Provide Opportunities to Empower and Support Girls and Women
Youth Events	• Promote Social Norms that Protect Against Violence<br>• Teach Skills to Prevent Sexual Violence<br>• Create Protective Environments
Blog posts	• Promote Social Norms that Protect Against Violence<br>• Teach Skills to Prevent Sexual Violence

Supplemental Appendix B

Monsoon RPE program

Agenda for meeting with youth staff

Sept 25 2023

Agenda

- Introductions
- Youth staff vision and dreams for the RPE program (open ended questions)
    - From your perspective, what is the goal of the work/activities you are doing with the RPE program at Monsoon?
        - How can this goal be achieved?
    - What excites you about this program?
    - In an ideal world, what changes would you like to see as a result of these activities?
    - What is one of your favorite activities that you have carried out? What made it your favorite?
    - Can you share a story from your work on this project that stands out to you/touched you/stayed with you?
    - What have you gained yourself from your work with this program? (knowledge, skills, social connections, etc)
- Review and discuss/get input into the logic model
- Review the table of activities linked to logic model
- AOB
- Next steps

Supplementary Appendix C: Complete table of Training activities implemented with Youth Leaders and Staff (YLS) linked to YLS logic model

How are Monsoon RPE Youth Leadership Training Activities linked to basic determinants in the Youth Leaders Logic Model?

Instructions: For each training activity, list the level of determinant (basic, intermediate, immediate) from the youth leaders logic model that it is ***DIRECTLY*** trying to impact, and explain how.

Note: Refer to Youth Leaders Logic Model & use exact words

Part I – Learning of YLS – activities involved in their growth as youth leaders and staff

**RPE Youth Leadership Training Activity (Current)**	**Youth Leaders Logic Model (Determinants w/ direct impact)**	**How does it do so?**
Training and Orientation	**Basic:** Learn about factors that lead to AAPI youth sexual violence (increase in knowledge)<br><br>**Intermediate:** Understand the nuance and complexity of issues	Orientation for youth staff covers an overview of Monsoon's background, core values, staff roles, programs, the AAAPI communities we serve, and a glimpse into the history of AAPI migration in the United States. Additionally, they receive training on sexual assault, domestic violence, and their prevalence within AAAPI communities.

	e.g., impact of gender and societal norms	Input from youth staff shapes their training to align with their specific roles and interests during the program. These trainings are also applicable and valuable outside of their work within Monsoon.
Background Reading	**Basic:** Learn about factors that lead to AAPI youth sexual violence (increase in knowledge), and Learn about current youth issues.<br><br>**Intermediate:** Understand the nuance and complexity of issues e.g., impact of gender and societal norms	Background reading on topics like teen dating violence, mental health in AAAPI youth, and other relevant information to the planning and implementation of their projects allows for them to provide accurate information and resources to peers.<br><br>This activity also encourages them to explore and learn more about their cultural backgrounds as Asian youth and how these identities intersect in their work.

Group Meetings	**Basic:** Create a safe non-judgmental space, and Be open to listening to new ideas<br><br>**Intermediate:** Bonding with peers, Feeling of belonging, and Express identity<br><br>**Immediate:** Feeling of solidarity/togetherness, and Feeling empowered	Team meetings allow for youth leaders to collaborate, problem-solve, and stay updated on each other's projects. Additionally, these meetings offer an opportunity to nurture and strengthen relationships, which fosters a more unified team.
Individual Check-ins	**Basic:** Create a safe non-judgmental space, Be willing to be vulnerable, and Be aware and	Engaging in one-on-one check-ins allows the Youth Coordinator to foster individual connections with each youth intern, addressing their specific needs, concerns, and expectations. These sessions also offer a space for sharing personal stories, challenges, and general updates beyond their work tasks.

	conscious of self-care (mental health)	
VPP Podcast	**Basic:** Be willing to be vulnerable<br><br>**Intermediate:**<br><br>Share knowledge with others that helps them understand and digest, Express identity, embrace who you are, and your culture, and take initiative/ability to speak out more to address uncomfortable topics	Platforms like podcasts provide a space for youth staff to have open conversations about topics relevant and important to youth such as the expectations and challenges of young Asian girls, dating, and social media activism. During these discussions they are also sharing their thoughts and experiences which allow them to connect with peers but also with each other.
Community Assessment & Evaluations of Program Impact	**Basic:** Be open to listening to new ideas	Evaluations play a crucial role in improving youth programs, allowing us to track self-performance, direct and long-term impact, and overall growth of our program. A prime example of this is the community assessment of the

	**Immediate:** Promote change in ways that are true to ourselves, and Feeling of accomplishment	Violence Prevention Team conducted by youth staff and the youth coordinator in 2021, which gathered feedback from past event participants, and insights from former interns. These findings provided information on the effectiveness of our programs among the youth we serve and those who have directly collaborated with Monsoon.

Part II - Implementing Programs - *YLS are main implementers*

**RPE Youth Leadership Training Activity (Current)**	**Youth Leaders Logic Model (Determinants w/ direct impact)**	**How does it do so?**
Implementation of Youth Programming	**Basic:**<br><br>Create a non-judgmental space, and Meet new people<br><br>**Intermediate:**	The Implementation of youth programming is centered around fostering an environment that encourages and empowers youth leaders to take charge and be proactive in their roles. It involves providing them with the space and opportunities to take the lead in their projects, while integrating their individual interests and passions.

	Identity Building, and Model a safe space in activities with others<br><br>**Immediate:**<br><br>Gain facilitation and time management skills, Gain Leadership Skills, and Reach other youth	This serves as a foundation for empowering them not only to navigate and manage their responsibilities but also to grow as confident leaders, role models, and advocates.
Project Planning/Concept Plans	**Immediate:** Gain facilitation and time management skills, Feeling of accomplishment, and Support individual passion	Youth staff create concept plans for their projects, detailing background information, discussion questions, target demographics, timelines, and budgets. Project planning also includes outreach and collaboration with other youth staff, and youth leaders in the schools and the community.
Youth Circles and Presentations	**Intermediate:** Share knowledge with others that helps them understand and digest	Facilitation of youth circle events, after-school groups, presentations at schools and organizations empowers them to take initiative and assume leadership roles. These events and presentations promote discussions on

	**Immediate:**<br><br>Gain facilitation and time management skills, Gain confidence, Gain leadership skills, and Reach other youth	healthy relationships in teens, the importance of wellness, stigma in AAAPI communities, and their work within the program.

Supplementary Appendix D: Evaluation research questions related to the Youth Leaders and Staff Logic Model assessment

Q#	Quantitative Research Question	Qualitative Research Question
Main evaluation questions		
1	a. To what extent do the activities implemented with youth staff address (map onto) the immediate, intermediate and basic determinants – as well as the outcomes - of the youth staff logic model?	b. How do the activities implemented with youth staff address the immediate, intermediate and basic determinants- as well as the outcomes - of the youth staff logic model? (What specific features of the activities made a difference/are necessary to achieve address logic model components?)
2	a. To what extent do the activities implemented with youth staff address (map onto) the risk/protective factors of the Iowa State RPE logic model?	b. How do the activities implemented with youth staff address (map onto) the risk/protective factors of the Iowa State RPE logic model? (What specific features of the activities made a difference/are necessary to achieve address logic model components?)
3	a. To what extent do the activities implemented with youth staff prepare them to conduct the activities with the public (the cascade to the activities youth staff carry out)?	b. How do the activities implemented with youth staff prepare them to conduct the activities with the public? (What specific features of the activities made a difference/are necessary to achieve address logic model components?)

4	a. To what extent do the activities implemented with youth staff lead to them being less likely to be victims or perpetrators of gender-based violence?	b. how do the activities implemented with youth staff lead to them being less likely to be victims or perpetrators of gender-based violence? (What specific features of the activities made a difference/are necessary to achieve address logic model components?)
5	a. To what extent is AAPI identity/culture imbedded in the activities implemented with youth staff?	b. How is AAPI identity/culture imbedded in the activities implemented with youth staff? (What specific features of the activities made a difference/are necessary to achieve address logic model components?)<br>c. Why is important that culture is imbedded in activities to be able to reach RPE outcomes?
6	(there is not equivalent quantitative question)	a.What lessons have been learned that might influence the types of activities implemented with youth staff?<br>b. How has the youth staff program impacted youth staff? How has it changed them? (might be linked to Q. 1b, 2b, 4b)<br>c. What stories illustrate the impact of the program on youth staff?
Additional evaluation questions of interest to YLS and Monsoon's ED:		

		i. What are the underlying assumptions behind the establishment of the youth staff program?<br>ii. How are youth staff selected? Does the characteristic of youth staff influence the extent to which the logic model is addressed? Is there an interaction between the selection of youth staff and possible outcomes of the youth staff program?<br>iii. How do the youth staff want to see the program grow? How do Monsoon leadership want to see the program grow?<br>iv. What is needed to sustain the program?

Supplementary Appendix F
Monsoon Asians & Pacific Islanders in Solidarity: _Staff Interview Protocols_

Set up

If the interview is in-person, please arrive at the location 30 minutes prior. For virtual interviews, sign-on 30 minutes prior to set up.

Introduction

- [Introduce the purpose of the interview.]
  My name is --. And thank you for coming today. Before we begin our discussion, I would like to say a few words about our purpose here today. This interview is a part of assessments being conducted by Monsoon to evaluate their Youth Violence Prevention Program (YVPP, and specifically the Youth Staff Program. The purpose of this interview is to improve the overall functioning/effectiveness of Monsoon's YVPP/RPE youth staff program. This will provide us with an enhanced understanding of what program elements are working well/as planned, and which areas are in need of improvement/additional efforts.
  I will ask you some specific questions, but the most important part of the discussion will be the information that you provide. We value your thoughts, beliefs, and experiences and are grateful that you're willing to share them with us. **My main goal is to learn from you and to have you feel comfortable during our conversation. Please know that we are coming to you as the expert in these matters and that there are no right or wrong answers to these questions.**

- [Informed Consent and Reportable Information]
  We would like to record our discussion, because we want to make sure we don't miss anything you share with us. We want to assure you that all of your comments are confidential and that nothing you say will be connected with your name or shared with any of your colleagues. Participation in this study is completely voluntary and will not affect your employment in any way. We will be the only ones with access to the tapes and their content. At the end of the study, we will erase the tapes and throw them away. If your prefer we do not need to record the session and during today's session you can ask me to turn the tape recorder off any time.

- [Distribute copies of consent statements, read aloud, ask the participant to sign the form, and collect it.]
  We expect this conversation to last for thirty minutes to an hour. Please feel free to get up at any time to help yourself to refreshments and to take a break if needed. The bathrooms are located ________________.

  Do you have any questions or concerns for me before we begin?
  Do we have your permission to start recording?

START RECORDING

- We are going to start recording now. Ok I will get started. Here is the youth staff logic model for reference as we talk through the questions. I'll give you a minute to look over the

immediate, intermediate, and basic determinants that staff and youth leaders identified as specific to the Youth Staff VPP.

QUESTIONS

1. What are the underlying assumptions behind the establishment of the YVPP youth staff program?
2. How are youth staff selected?
3. In the youth staff program, what do the youth leaders learn about Gender-Based Violence (GBV)?
4. What activities do you believe best address GBV prevention?
5. How are AAPI identities embedded in the activities conducted with the youth leaders?
6. Why do you think it's important for cultural elements to be embedded in activities?
    a. *Can you provide examples of activities that have been particularly effective in this regard?*
7. In the survey, it was identified that youth believed XX activities were most associated with XX outcomes for the youth leaders. Can you share specific examples of how you think these activities contributed to achieving the outcomes?
    a. *For example: How did training and orientation increase the youth leader's sense of belonging and solidarity?*
8. Across all surveys, X goal was never selected. Why do you think that is? Are there better ways we could be addressing X?
9. How do the activities in the youth staff program reduce the likelihood of the youth leaders becoming victims or perpetrators of gender-based violence?
10. How do you think the activities the youth leaders participate in prepare them to conduct similar activities with the public and other youth?
11. In your opinion, what features of the activities are most necessary to effectively prepare them for conducting activities with the public?
12. What is your favorite activity they conduct with the other youth? Why?
13. When they've gone out into the public, have there been any unexpected challenges? How can we change the youth leader activities to address these challenges?
14. What lessons have been learned about the process or outcomes of the YVPP in the years it has been implemented?
15. What types of activities would you like to see implemented with youth staff in the future?
16. Could you share any stories or anecdotes that illustrate the impact of the program on you or the youth you've interacted with?
17. What would you like to change about the program?
18. How do you want to see the program grow?
19. What is needed to sustain the program?
20. Is there anything else you'd like to share with us about the YVPP that we have not already asked?

Thank you for sharing your thoughts with us. We will be developing a report to share with you that summarizes the findings of all evaluation assessments.

Monsoon Asians & Pacific Islanders in Solidarity: *Youth Leader Focus Group Interview Protocols*

Preparation (specific for in-person sessions)

At each focus group, the facilitators should bring the following supplies:

- Name tags (cardboard for nameplate/pronouns and markers)
- Two tape recorders, 2-3 tapes (120 minutes), and enough batteries
- Notepad and pens
- Consent forms
- Monsoon materials (if applicable)
- Human participant consent informational sheet (if applicable)
- Referral information sheet
- Refreshment/Lunch
- Token gifts
- Tissues
- Flip charts and markers

Set up

If the FGD is in-person, please arrive at the location 30 minutes prior to the Focus Group. For virtual FGD, sign-on 30 minutes prior to set up.

2. Set up table and put chairs around it.
3. Put the main facilitator's nameplate on the table in front of a chair that is right across a wall clock to monitor timing of the focus group, and the assistant facilitator should sit across from the facilitator so that two of you can clearly see all the participants.
4. Check tape-recording
5. Place tissue boxes on the table
6. Put cardboard for nameplates and markers on the table
7. Set up refreshment table in the corner of the room
8. Set up tape recorders, one at the main facilitator's seat, and the other at the assistant facilitator's seat.

What facilitators should do as participants arrive

Welcome participants, thank them for coming, and show them the nameplates, and the refreshment table.

- [Upon arrival participants will create nameplates with their first name or a name they prefer to be called.]
- My name ----. Thank you for joining us today. Please sit wherever you would like to and have some refreshments while we are waiting for other participants.
- [If some participants did not arrive by the assigned time for the focus group to start, wait for 10 minutes, and let other participants know that the group will start in 10 minutes.]
- Some participants have not arrived yet. We will wait for 10 more minutes and start the focus group regardless of their arrival.

Introduction

- [Introduce Monsoon and the purpose of the focus group.]

My name is --. And thank you for coming today. Before we begin our discussion, I would like to say a few words about our purpose here today. This focus group is a part of assessments being conducted by Monsoon to evaluate their Youth Violence Prevention (RPE) Program (YVPP), and specifically the Youth Staff Program which you were/are a part of. The purpose of this focus groups is improve the overall functioning/effectiveness of Monsoon's YVPP/RPE youth staff program. This will provide us with an enhanced understanding of what program elements are working well/as planned, and which areas are in need of improvement/additional efforts. I will ask you some specific questions, but the most important part of the discussion will be the information that you provide.

We value your thoughts, beliefs, and experiences and are grateful that you're willing to share them with us. My main goal is to learn from you and to have you feel comfortable during our conversation. Please know that we are coming to you as the expert in these matters and that there are no right or wrong answers to these questions.

- [Informed Consent and Reportable Information]

We would like to first ask for your permission to participate in this focus group. We will also be tape recording our time together so that we can remember what was said in the discussion. We will be the only ones with access to the tapes and their content. At the end of the study, we will erase the tapes and throw them away. During today's session you can ask me to turn the tape recorder off any time. If your prefer we do not record the session, let me know and we can just take notes. You can also ask me to turn off the recording at any time.

- [Distribute copies of consent statements to participants, read aloud to the group, ask each participant to sign the forms, and collect them.]

- [Introduce Roles of Focus Group Staff]

To make the discussion go smoothly, my role as a facilitator will be to ask questions, clarify your answers when necessary, and keep us focused on the major questions. My colleague (name) will be taking notes for us just in case the recording does not work.

- [Discuss Timing of Focus Group]

We expect this conversation to last for an hour to an hour and a half. Please feel free to get up at any time to help yourself to refreshments and to take a break if needed. The bathrooms are located _.

- [Group Introductions/Ice breaker]

Let's do a group introduction. We would like to start by asking each of you to tell us your first name, or what you would prefer to be called, and something that is new and good in your life. It may be something small, or something big.... I will begin ---- y name is....

- Each participant should then introduce themself.

- [Discussion Guidelines]

To make this Focus Group comfortable for everyone, we would like to make suggestions to help guide our conversation (put the guidelines on the wall):

- This conversation should be kept confidential. In other words, whatever is discussed here should not leave the room.

- Please speak up --- please do not hesitate to share your ideas and opinions.
- Please do not interrupt other participants. If you wish to speak while another participant is speaking, please quietly raise your hand. We will make sure you have an opportunity to speak.
- There are no right or wrong answers to the questions we will ask you. Rather, we are interested in understanding your opinions and ideas.
- Please share your opinions with one another so we can build off each other's ideas.
- Please pay attention and listen to what other people have to say.
- Please do not mention a specific person's name
- Agree to disagree.

Do you have questions, comments, suggestions on the discussion guidelines?

- Ask if they have questions.

Do you have any questions before we begin?

START RECORDING

We are going to start recording now. Ok I will get started. Here is the youth staff logic model for reference as we talk through the questions. I'll give you a minute to look over the immediate, intermediate, and basic determinants that staff and youth leaders identified as specific to the Youth Staff VPP.

QUESTIONS

We're going to start off asking you to expand on some of the results we saw in the survey you filled out:

1. In the survey, it was identified that you believed XX activities were most associated with XX outcomes. Can you share specific examples of <u>how</u> these activities contributed to achieving the outcomes?
    a. *For example: How did training and orientation increase your sense of belonging and solidarity?*

21. Could you share any stories or anecdotes that illustrate how X changed your perspective on X?
22. Were there any aspects of X activity that could be strengthened to better meet the goal of X?
23. Across all surveys, X goal was never selected. Why do you think that is? Are there better ways we could be addressing X?
24. Could you share any stories or anecdotes that illustrate how you've implemented X in your daily life?

We have heard about the importance of considering the AAPI identity in the activities of Monsoon.

25. How is your AAPI identity embedded in the activities you participate in as youth leaders?
26. Why do you think it's important for cultural elements to be embedded in activities?
27. How have these experiences impacted your view of your own relationship with your culture and identity?

We are now going to transition to some questions about how your knowledge and beliefs about GBV have been affected by your participation in the YVPP as a youth staff:

28. What have you learned about GBV through your time as a youth leader?
29. What activities do you believe best address GBV?

30. Have you seen real world examples of GBV? Is this something that you had noticed before?
31. How do the youth staff activities you've participated in reduce the likelihood of becoming a victim or perpetrator of gender-based violence?
    a. *Can you provide examples of activities that have been particularly effective in this regard?*

Now we'd like to ask you some questions about how the activities you received as a youth staff prepared you to conduct the activities with the public.

32. How do the activities you've participated in as youth leaders prepare you to conduct similar activities with the public and other youth?
33. In your opinion, what features of the activities are most necessary to effectively prepare you for conducting activities with the public?
34. What has been your favorite activity that you've conducted with other youth? Why?
35. When you've gone out into the public, have there been any unexpected challenges? How can we change the youth leader activities to address these challenges?

Finally, this set of questions is about how you have been impacted by being a youth leader in the YVPP and how you'd like to see this program grow:

36. What lessons have you learned during your time as a youth leader?
37. What types of activities would you like to see implemented with youth staff in the future?
38. How has participation in the youth staff program impacted you outside of the program? Can you share any changes or developments you've seen in your daily life?
39. Could you share any stories or anecdotes that illustrate the impact of the program on you or the youth you've interacted with?
40. What changes would you like to see made in the youth staff VPP activities? Or processes?
41. How would you like to see the program grow?
42. Is there anything else you think is important for us to know about the YVPP?

Thank you for sharing your experiences with us. We will be developing an evaluation report that synthesizes the results of all the assessment tools, which will be shared with you. Do you have any final questions for us?

IOWA

Demographics

Q1. We are inviting you to participate in a survey about your experiences as a Youth Leader with Monsoon. This survey will take approximately 15 minutes. Your responses are anonymous, and you may skip any questions that you do not wish to answer.

Thank you for your time and participation.

Q2. Do you consent to participate in this survey and allow us to use your responses for research purposes only?

- ○ Yes
- ○ No, I do not wish to participate in this survey

Q3. How old are you in years?

Q4. What grade are you in?

- 9th (Freshman)
- 10th (Sophmore)
- 11th (Junior)
- 12th (Senior)
- GED
- Graduated from High School
- Currently in a 4 year or community college

Q5. How do you describe your gender identity?

- Male
- Female
- Non-binary / third gender
- Transgender
- Prefer not to respond

Q6. How would you describe your cultural identity?

- ◯ Add identity options here

Q7. What is the main language you use at home?

- ◯ Add language options here

Q8. Were you born outside the United States?

- ◯ Yes
- ◯ No

Q9. At what age (in years) did you immigrate to the US?

[ ]

Q10. What month and year did you first start as a youth leader with Monsoon? Please provide your best estimate.

Month (mm) ☐

Year (yyyy) ☐

Default Question Block

Q11. The following questions are related to the activities that you may have participated in with Monsoon as a youth leader:

Q12. Of the following activities offered to youth leaders, which did you participate in? (Select all that apply)

- ☐ Training and Orientation
- ☐ Background Readings
- ☐ Group Meetings
- ☐ Individual Check-ins
- ☐ VPP Podcast
- ☐ Community Assessment & Evaluations of Program
- ☐ Implementing Youth Programming
- ☐ Project Planning
- ☐ Youth Circles and Presentations

Q13. Of the activities you participated in, which was your favorite?

- » Training and Orientation
- » Background Readings
- » Group Meetings
- » Individual Check-ins
- » VPP Podcast
- » Community Assessment & Evaluations of Program
- » Implementing Youth Programming
- » Project Planning
- » Youth Circles and Presentations

Q14. We are interested in how your participation in these activities changed or grew your skills and perspectives. For each activity you participated in as a youth leader, please select up to three items you believe were most impacted by this activity.

	» Training and Orientation	» Background Readings	» Group Meetings	» Individual Check-ins	» VPP Podcast	» &
Feeling of Belonging/Solidarity	☐	☐	☐	☐	☐	

	» Training and Orientation	» Background Readings	» Group Meetings	» Individual Check-ins	» VPP Podcast	» &
Leadership/facilitation skills	☐	☐	☐	☐	☐	
Understanding the nuance and complexity of issues	☐	☐	☐	☐	☐	
Expressing identity, embracing who you are and your culture	☐	☐	☐	☐	☐	
Being a role model/positive influence	☐	☐	☐	☐	☐	
Taking initiative to speak out and address uncomfortable topics	☐	☐	☐	☐	☐	
Sharing knowledge with others	☐	☐	☐	☐	☐	
Gaining confidence	☐	☐	☐	☐	☐	
Feeling accomplished/empowered	☐	☐	☐	☐	☐	
Visibility and representation of the API community	☐	☐	☐	☐	☐	
Inspiring the next generation	☐	☐	☐	☐	☐	
Collaborative activism	☐	☐	☐	☐	☐	
None of the above	☐	☐	☐	☐	☐	

API Culture

Q15. Please rate the extent to which you agree or disagree with the following statements about the activities implemented with youth staff: "These activities..."

	Strongly disagree	Somewhat disagree	Neither agree nor disagree	Somewhat agree	Strongly agree
Celebrate and honor API identity and culture	○	○	○	○	○
Provide opportunities for sharing diverse experiences, histories and traditions	○	○	○	○	○
Incorporate elements such as language, cuisine, music, or art specific to API cultures	○	○	○	○	○
Provide an inclusive space for discussing API issues	○	○	○	○	○
Have helped me develop a stronger connection to my API identity and cultural heritage	○	○	○	○	○

Block 2

Q16. Please rate the extent to which your time as a youth leader with Monsoon has impacted your confidence in the following:

	Less confident	Neither more or less confident	More confident
Expressing your identity and embracing your culture	○	○	○
Taking the initiative to speak out and adress uncomfortable topics	○	○	○
Engaging in advocacy and activism	○	○	○
Sharing knowledge with others in a way that helps them understand	○	○	○

Q17. Are there any activities or trainings you wish were provided?

Activities Led

Q18. The following questions related to the activities you have led, or will be leading with the community.

Q19. Which events have you already led, or think you will be leading in the future? Select all that apply.

- ☐ Youth Circle Events
- ☐ The Brunch Club
- ☐ Through Your Lens
- ☐ Let's Talk About It
- ☐ API Student Association
- ☐ Boys Group
- ☐ Girls Group
- ☐ None of the above

Q20. Of these events, which are you most excited about?

- ○ » Youth Circle Events
- ○ » The Brunch Club
- ○ » Through Your Lens
- ○ » Let's Talk About It

- ○ » API Student Association
- ○ » Girls Group
- ○ » Boys Group
- ○ » None of the above

Q21. We are interested in how the activities you have participated in as a youth leader have prepared you to lead activities with the community.

For each youth leader activity please select which community activities it best prepared you to lead/facilitate.

	» Training and Orientation	» Background Readings	» Group Meetings	» Individual Check-ins	» VPP Podcast	» Community Assessment & Evaluations of Program
» Youth Circle Events	☐	☐	☐	☐	☐	☐
» The Brunch Club	☐	☐	☐	☐	☐	☐
» Through Your Lens	☐	☐	☐	☐	☐	☐
» Let's Talk About It	☐	☐	☐	☐	☐	☐

	» Training and Orientation	» Background Readings	» Group Meetings	» Individual Check-ins	» VPP Podcast	» Community Assessment & Evaluations of Program
» API Student Association	☐	☐	☐	☐	☐	☐
» Girls Group						
» Boys Group	☐	☐	☐	☐	☐	☐
» None of the above	☐	☐	☐	☐	☐	☐
	☐	☐	☐	☐	☐	☐

Q22. Are there any activities that you feel you need more training on to confidently lead?

☐ Youth Circle Events

☐ The Brunch Club

☐ Through Your Lens

☐ Let's Talk About It

☐ API Student Association

☐ Girls Group

☐ Boys Group

☐ None of the above

GBV

Q23. Monsoon's mission is "to end all forms of gender-based violence (GBV) and build healthy communities through transformative justice and social change". We are interested in how your time as a Monsoon youth leader has impacted your knowledge, beliefs and perceptions about GBV.

Q24. In your own words, how would you define "Gender Based Violence"?

Q25. Please rate the extent to which you agree or disagree with the following statements: "Since working as a youth leader with Monsoon, I..."

	Strongly disagree	Somewhat disagree	Neither agree nor disagree	Somewhat agree	Strongly agree
Am more knowledgeable about GBV	○	○	○	○	○
Better understand the causes of GBV	○	○	○	○	○
Am more likely to intervene if I witness an incident of GBV	○	○	○	○	○
Am less likely to become a perpetrator of GBV	○	○	○	○	○
Am less likely to become a victim of GBV	○	○	○	○	○

Q26. Of the activities you participated in as a youth leader, which increased your knowledge about GBV? (Select all that apply)

- ☐ None
- ☐ » Training and Orientation
- ☐ » Background Readings
- ☐ » Group Meetings
- ☐ » Individual Check-ins
- ☐ » VPP Podcast
- ☐ » Community Assessment & Evaluations of Program
- ☐ » Implementing Youth Programming

- [ ] » Project Planning
- [ ] » Youth Circles and Presentations

Q27. Of the activities you participated in as a youth leader, which had an impact on your beliefs surrounding GBV? (Select all that apply)

- [ ] None
- [ ] » Training and Orientation
- [ ] » Background Readings
- [ ] » Group Meetings
- [ ] » Individual Check-ins
- [ ] » VPP Podcast
- [ ] » Community Assessment & Evaluations of Program
- [ ] » Implementing Youth Programming
- [ ] » Project Planning
- [ ] » Youth Circles and Presentations

Q28. Of the activities you participated in as a youth leader, which had increased your confidence to intervene on GBV? (Select all that apply)

- [ ] None
- [ ] » Training and Orientation
- [ ] » Background Readings

- ☐ » Group Meetings
- ☐ » Individual Check-ins
- ☐ » VPP Podcast
- ☐ » Community Assessment & Evaluations of Program
- ☐ » Implementing Youth Programming
- ☐ » Project Planning
- ☐ » Youth Circles and Presentations

Q29. Of the activities you participated in as a youth leader, which decreased your probability of becoming a perpetrator of GBV? (select all that apply)

- ☐ None
- ☐ » Training and Orientation
- ☐ » Background Readings
- ☐ » Group Meetings
- ☐ » Individual Check-ins
- ☐ » VPP Podcast
- ☐ » Community Assessment & Evaluations of Program
- ☐ » Implementing Youth Programming
- ☐ » Project Planning
- ☐ » Youth Circles and Presentations

Q30. Of the activities you participated in as a youth leader, which had an impact on your ability to protect yourself from becoming a victim of GBV? (select all that apply)

- ☐ None
- ☐ » Training and Orientation
- ☐ » Background Readings
- ☐ » Group Meetings
- ☐ » Individual Check-ins
- ☐ » VPP Podcast
- ☐ » Community Assessment & Evaluations of Program
- ☐ » Implementing Youth Programming
- ☐ » Project Planning
- ☐ » Youth Circles and Presentations

Q31. Are there any domains of GBV you believe are not being thoroughly discussed, or that you still have questions about?

[ ]

Powered by Qualtrics

Supplementary Appendix (SA) G

Guiding the evaluation of the Monsoon RPE program: Preliminary measures/indicator, data source/data collection instrument, audiences, timing of evaluation, audience, and data analysis for each evaluation question

**Evaluation question**	**Measure or indicator**	**Data source/Data collection instrument**	**How often**	**Participants**	**Data Analysis**
1A	Extent to which participants rate each activity as linked to the Youth Staff Program Logic Model (LM) determinants and outcomes	Survey – for each activity, choose from drop down menu of determinants and outcomes (SA C, Q.14-16 / Q.13 related)	Every other year	✓ Current and future youth staff<br>✓ Monsoon Leadership	Frequencies
1B	Themes from Focus Group Discussion (FGDs) and interviews	FGDs with *youth staff* (Appendix D, Q. 1-4 / Q.5 related)<br>Interviews with *Monsoon Leadership* (Appendix D, Q.7-8)	Every 3rd year or at times of significant change to program	✓ Current and future youth staff<br>✓ Past youth staff<br>✓ Monsoon Leadership	Thematic analysis
2A	Extent to which participants rate each activity as linked to risk/protective factors of the Iowa State RPE logic model	Survey – for each activity, choose from drop down menu of risk/protective factors (Appendix C, Q. 25-30)	Every other year	✓ Current and future youth staff<br>✓ Monsoon Leadership	Frequencies
2B	Themes from FGDs and interviews	FGDs with *youth staff* (Appendix D, Q.9, 12)<br>Interviews with *Monsoon Leadership* (Appendix D, Q.9)	Every 3rd year or at times of significant change to program	✓ Current and future youth staff<br>✓ Past youth staff<br>✓ Monsoon Leadership	Thematic analysis
3A	Extent to which participants rate each activity as important for their preparing to conduct the activities to the public (the	Survey – for each activity, choose from drop down menu of cascading activities to the	Every other year	✓ Current and future youth staff	Frequencies

	cascade to the activities youth staff carry out)	public/community (Appendix C, Q.21 / Q.19-20,22 related)		✓ Past youth staff<br>✓ Monsoon Leadership	
3B	Themes from FGDs and Interviews	FGDs with *youth staff* (Appendix D, Q/13-14 / 15-16 related)<br>Interviews with *Monsoon Leadership* (Appendix D, Q.10-11 /13-14 related)	Every 3rd year or at times of significant change to program	✓ Current and future youth staff<br>✓ Past youth staff<br>✓ Monsoon Leadership	Thematic analysis
4A	Extent to which participants rate each activity as linked to youth staff being less likely to be a perpetrator or victim of gender-based violence	Survey questions (Appendix C, Q.29-30)	Every other year	✓ Current and future youth staff<br>✓ Past youth staff	Frequencies
4B	Themes from FGDs	FGDs with *youth staff* (Appendix D, Q.12)<br>Interview with *Monsoon Leadership* (Appendix C, Q.9)	Every 3rd year or at times of significant change to program	✓ Current and future youth staff<br>✓ Past youth staff	Thematic analysis
5A	Extent to which AAPI culture is imbedded in each activity	Survey questions (Appendix C, Q.15- 16)	Every other year	✓ Current and future youth staff<br>✓ Monsoon Leadership	Frequencies
5B and 5C	Themes from FGDs and Interviews	FGD with *youth staff* (Appendix D, Q. 6-8)<br>Interviews with *Monsoon Leadership* (Appendix D, Q.5-6)	Every 3rd year or at times of significant change to program	✓ Current and future youth staff<br>✓ Monsoon Leadership	Thematic analysis
6 A-C	Themes from FGDs and Interviews	FGD with *youth staff* (Appendix D, Q.17,19,20)	Annually	✓ Current and future youth staff	Thematic analysis

		Interviews with *Monsoon Leadership* (Appendix D, Q.14-16)		✓ Past youth staff<br>✓ Monsoon Leadership	
Extra questions i-iv	Themes from Interviews	Interviews with *Monsoon leadership* (Appendix D, Q. 18-19)	Once and at intervals where significant changes to program occur	✓ Monsoon Leadership	Thematic analysis
Extra question iii and iv	Themes from FGD	FGD with *youth staff* (Appendix F, Q.17,22)	Every other year	✓ Current youth staff	Thematic analysis

*https://usaidlearninglab.org/sites/default/files/resource/files/27-mod5_definingevaluationquestions.pdf
